# Model-Free Transformer Framework for 6-DoF Pose Estimation of Textureless Tableware Objects

**DOI:** 10.3390/s25196167

**Published:** 2025-10-05

**Authors:** Jungwoo Lee, Hyogon Kim, Ji-Wook Kwon, Sung-Jo Yun, Na-Hyun Lee, Young-Ho Choi, Goobong Chung, Jinho Suh

**Affiliations:** 1 Smart Mobility Research Center, Korea Institute of Robotics and Technology Convergence (KIRO), Pohang 37666, Republic of Korea; ricow@kiro.re.kr (J.L.); hgkim@kiro.re.kr (H.K.); jwkwon@kiro.re.kr (J.-W.K.); yunsj@kiro.re.kr (S.-J.Y.); nahyun7373@kiro.re.kr (N.-H.L.); rockboy@kiro.re.kr (Y.-H.C.); goobongc@kiro.re.kr (G.C.); 2 Major of Mechanical System Engineering, Pukyong National University, Busan 48513, Republic of Korea

**Keywords:** 6-DoF pose estimation, transformer encoder architecture, geometry-based features, surface normal vector, tableware manipulation

## Abstract

Tableware objects such as plates, bowls, and cups are usually textureless, uniform in color, and vary widely in shape, making it difficult to apply conventional pose estimation methods that rely on texture cues or object-specific CAD models. These limitations present a significant obstacle to robotic manipulation in restaurant environments, where reliable six-degree-of-freedom (6-DoF) pose estimation is essential for autonomous grasping and collection. To address this problem, we propose a model-free and texture-free 6-DoF pose estimation framework based on a transformer encoder architecture. This method uses only geometry-based features extracted from depth images, including surface vertices and rim normals, which provide strong structural priors. The pipeline begins with object detection and segmentation using a pretrained video foundation model, followed by the generation of uniformly partitioned grids from depth data. For each grid cell, centroid positions, and surface normals are computed and processed by a transformer-based model that jointly predicts object rotation and translation. Experiments with ten types of tableware demonstrate that the method achieves an average rotational error of 3.53 degrees and a translational error of 13.56 mm. Real-world deployment on a mobile robot platform with a manipulator further validated its ability to autonomously recognize and collect tableware, highlighting the practicality of the proposed geometry-driven approach for service robotics.

## 1. Introduction

Demand for service robots is growing, especially in restaurants, where labor shortages and the need for contactless service have become more urgent due to demographic changes and concerns about infection control. Service robots can assist with tasks such as food delivery, dish collection, and cleaning. This reduces human contact and improves operational consistency. However, existing delivery robots usually require human assistance for loading and unloading, which limits full automation. The ability to manipulate tableware autonomously using a robotic arm is a critical step toward achieving seamless robotic service in restaurants.

For such robotic manipulation, object pose estimation is essential. This technique determines an object’s three-dimensional position and orientation relative to a reference coordinate frame, such as the robot’s camera or base frame. Six-degree-of-freedom (6-DoF) object pose estimation is a fundamental computer vision task that involves determining the three-dimensional (3D) translation and rotation of objects from visual observations [1]. Due to its importance in robotics, augmented reality, autonomous driving, and industrial automation, this problem has been extensively studied [2,3].

Despite this research, estimating the pose of small-scale, textureless objects, such as plates, bowls, and cups, remains a critical challenge. Unlike industrial or commercial objects with rich surface patterns, tableware often has a uniform color and glossy or reflective surfaces with minimal textural features. This makes conventional, feature-based methods unreliable. The wide variability in shape and size across different categories of tableware also complicates pose estimation. Real-world dining environments introduce additional difficulties, such as partial occlusion, food residue on surfaces, and variable lighting, all of which reduce perception accuracy.

Accurate 6-DoF pose estimation allows the robot to identify where the object is located and how it is rotated in space, which is essential for tasks such as grasping, moving, and placing objects. In the context of robotic tableware collection, high positional and rotational accuracy is necessary to determine graspable regions within tight tolerances, typically within a few centimeters, to ensure safe and reliable manipulation. However, conventional deep learning-based pose estimation approaches often only estimate the overall object pose and may lack the precision necessary for reliable grasping. Furthermore, these models may not generalize well to tableware items with contaminated surfaces or color variations caused by food residue. To grasp objects consistently and reliably, it is necessary to extract stable grasp points across different instances of similar tableware, regardless of surface condition.

In this study, we propose a method for estimating the 6-DoF pose of tableware objects that utilize geometry-driven features and a transformer encoder to address the limitations of texture-dependent and CAD-based approaches. This method provides robust, model-free, texture-free, six-degree-of-freedom (6-DoF) pose estimation, making it ideal for practical service robotics applications. The pipeline begins with object detection and segmentation using a pretrained video foundation model that identifies tableware instances in color images and generates corresponding segmentation masks. From the aligned depth image, surface vertices within the masked region are extracted and divided into a uniform 2D grid. Surface normal vectors are then computed for each grid cell. These normal vectors, particularly those from the upper rim region, are used as input to a transformer-based deep learning model that infers the object’s position and orientation.

**Figure 1:**
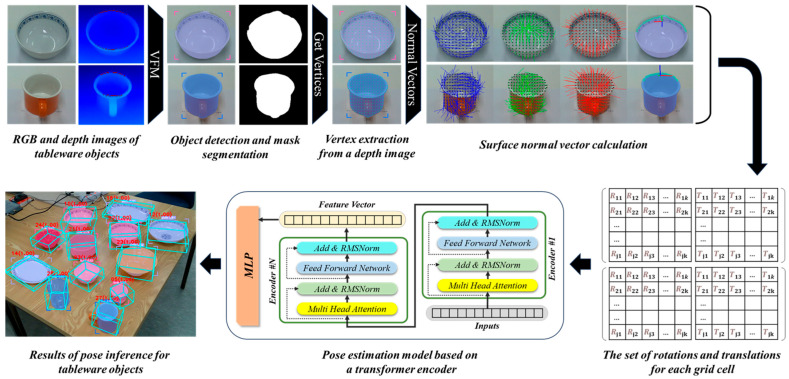
Proposed framework for 6-DoF pose estimation of tableware objects: detection and segmentation from RGB images, vertex and surface normal extraction from depth data, transformer-based feature encoding, and final pose prediction for robotic manipulation.

The primary goal of this approach is to accurately estimate the pose of textureless tableware objects without relying on a 3D CAD model. This approach provides a consistent representation that enables the manipulation of objects in unstructured environments. Figure 1 illustrates the overall process of the proposed model-free and texture-free, 6-DoF pose estimation framework.

The contributions of this research are as follows:

- We propose a novel representation that partitions the surface vertices of tableware objects into a uniformly spaced 2D grid. The centroid position and surface normal vector are computed for each grid cell, and these features collectively serve as the input for the pose estimation model. Since surface normals directly encode the object’s orientation, this structured representation provides rich geometric information, enhancing the accuracy and efficiency of 6-DoF pose estimation. Unlike unstructured point cloud inputs, the grid-based format ensures a fixed input dimension, which is compatible with transformer encoders and avoids the need for padding or truncation strategies.
- A distinguishing characteristic of tableware objects is the presence of an open upper rim. In our approach, we explicitly extract rim-specific vertices from the segmented contour and incorporate them into the pose estimation process. The normal vectors of these rim vertices provide stable orientation cues that significantly reduce the impact of pose estimation errors caused by food residues or occlusions inside the container. By emphasizing rim geometry, our method leverages structural features that are consistent across diverse tableware categories such as bowls, cups, and plates.
- Unlike conventional approaches that rely on CAD models or color texture information, our method performs pose estimation using only depth information. This makes the system more robust in real-world conditions where tableware surfaces may be contaminated, reflective, or lack distinctive texture. Since our approach relies exclusively on geometry-based features, it can effectively generalize to shapes of tableware not used during training, eliminating the need for object-specific models.

## 2. Related Works

Object Detection. Deep learning has revolutionized object detection, enhancing accuracy and efficiency across various applications. This technology leverages convolutional neural networks (CNNs) to automate feature extraction, significantly outperforming traditional methods.

Deep learning methods primarily utilize CNNs, which can be categorized into one-stage and two-stage approaches. This architecture has shown remarkable improvements in detection performance [4].

A two-stage detector is a model that performs the object detection process in two stages. It begins by extracting regions (region suggestions) where objects may exist in the image and then classifying each extracted region into a class and predicting the correct bounding box. Two-stage detectors are well-suited for tasks demanding high precision in object detection. Region with CNN features (R-CNN) is a pioneering model that introduced the use of CNNs for object detection, employing Selective Search for region proposals [5]. Fast R-CNN Improve upon R-CNN by introducing ROI pooling for more efficient feature extraction [6].

Unlike two-stage detectors like Faster R-CNN, which generate region proposals in a separate stage before classification, one-stage detectors perform object detection in a single step. This means they directly predict both the bounding boxes and class probabilities for objects within the image. You Only Look Once (YOLO) is a state-of-the-art, real-time object detection system. YOLO treats object detection as a regression problem from image pixels to bounding box coordinates and class probabilities. This unified approach allows YOLO to make predictions with a single network evaluation [7]. Single Shot MultiBox Detector (SSD) directly predicts bounding boxes and class probabilities from the input image in a single forward pass. SSD uses multiple feature maps of different scales to detect objects of various sizes. This allows it to handle objects of different scales more effectively than YOLO, which primarily relies on a single feature map [8].

Traditional object detection models have been highly successful, but they require large amounts of labeled data and are limited to detecting predefined classes. Detection Anything models are a cutting-edge advancement in deep learning–based object detection. Unlike traditional models, which are trained on specific object classes, Detection Anything models aim to detect any object within an image or video, including objects not included in the training data. DINO (Distillation of Non-contrastive Image Representations) is a self-supervised learning method designed to train neural networks on large amounts of unlabeled image data. It has gained significant attention due to its simplicity and effectiveness, especially when combined with Vision Transformers (ViT) [9]. Grounding DINO is a cutting-edge model that integrates language and vision for open-set object detection, enabling the identification of arbitrary objects based on user-defined inputs. This model has shown significant advancements in various applications, particularly in zero-shot detection and automated image annotation [10]. YOLO-World is a novel framework that enhances YOLO with open-vocabulary object detection capabilities through vision-language pre-training. By leveraging a large-scale pre-trained vision-language model, it can detect objects beyond the predefined classes [11].

Segmentation. Image segmentation is the process of dividing a digital image into multiple regions that are meaningful and correspond to specific structural or semantic content. Unlike image classification, which assigns a single label to an entire image, segmentation provides pixel-level labeling, offering a higher level of detail. This allows for a more precise interpretation and analysis of visual scenes. For example, in object detection tasks involving dish recognition, segmentation techniques are used to accurately delineate object boundaries and extract individual tableware contours from an image. The U-Net architecture is popular for biomedical image segmentation and consists of a contracting path for feature extraction and an expansive path for precise localization [12]. Mask R-CNN combines instance segmentation with object detection, enabling the identification and segmentation of multiple objects within an image [13]. Meta AI’s groundbreaking model, Segment Anything, has significantly advanced the field of image segmentation [14]. Unlike traditional models designed for specific tasks or datasets, Segment Anything is a versatile model that can accurately segment arbitrary regions within images of various types and complexities.

Object Pose Estimation. Six-degree-of-freedom (6-DoF) object pose estimation is a key problem in computer vision that seeks to determine an object’s three-dimensional translation and rotation from visual data. Due to its critical role in fields such as robotics, augmented reality, autonomous driving, and industrial automation, it has been the subject of extensive research. Research in this field has progressed from early geometry-based approaches to advanced deep learning methods, which are generally categorized as either instance-level or category-level pose estimation.

Instance-level pose estimation requires prior knowledge of the target objects, typically in the form of CAD models, 3D meshes, or detailed geometric descriptions [15]. Early methods relied on feature matching and template-based techniques. In these methods, handcrafted descriptors, such as SIFT [16], were used to establish correspondences between 2D observations and 3D object models. Template-based approaches generated synthetic viewpoints and matched them against observed images to estimate pose [17]. The emergence of deep learning has significantly improved accuracy and robustness. Keypoint-based methods detect 2D image keypoints and establish correspondences with 3D model points to solve pose via Perspective-n-Point (PnP) [18]. CNN-based methods have enhanced this process. For example, PVNet introduced pixel-wise voting to improve robustness under occlusion [19]. Direct regression methods predict pose parameters directly from RGB input using deep neural networks. These methods include modified YOLO networks [20] and encoder–decoder convolutional neural networks [21]. Multi-task regression models predict multiple pose parameters simultaneously [22,23], while render-and-compare approaches, such as RMTrack [24] and NeRF-Pose [25], exploit synthetic rendering or neural radiance fields for robust or weakly supervised pose estimation.

Category-level pose estimation presents a more challenging paradigm by predicting object poses without requiring instance-specific CAD models. This approach enables generalization to unseen objects within known categories, which is particularly valuable in real-world applications where exhaustive modeling of all instances is impractical. Early approaches explored canonical shape learning and deformation fields, most notably through the Normalized Object Coordinate Space (NOCS) framework [26], which jointly estimates masks, canonical coordinates, and poses from RGB-D images. Recently, transformer architectures have been increasingly incorporated into category-level tasks. For example, YOPO [27] formulates 9-DoF estimation (pose plus size) as an extension of 2D object detection, employing query-based attention to fuse features and geometric priors. Similarly, RCGNet [28] uses transformer-based geometric guidance to achieve RGB-only category-level pose estimation without requiring depth at inference.

Multi-modal methods have advanced this field by integrating RGB, depth, and textual information. MK-Pose [29] introduces a multimodal keypoint learning framework that uses visual and semantic cues to improve generalization across categories. Additionally, HRC-Pose [30] uses contrastive learning to enforce geometric consistency in point cloud representations, thereby preserving pose continuity across different instances within the same category.

Textureless object pose estimation is a significant challenge in computer vision because it requires determining the 6-DoF of objects lacking sufficient texture, distinctive keypoints, or visual patterns. Since traditional texture-dependent methods rely on these features, specialized approaches based on geometric information, shape templates, or multimodal information are necessary Zhao et al. [31] proposed a two-phase deep learning method for estimating the pose of textureless objects using RGB-D images. Their framework consists of an initial pose approximation stage followed by iterative refinement with a point cloud network, achieving an average recognition rate of 98.3% on an industrial parts dataset. However, this approach relies heavily on RGB-D sensors, making it highly susceptible to depth noise, and the errors introduced by depth inaccuracies tend to accumulate through the iterative refinement process. Furthermore, the iterative refinement increases computational cost, which restricts its applicability in real-time robotic systems. Cao et al. [32] introduced a scalable, real-time framework for estimating the pose of textureless objects based on GPU-accelerated template matching. Their method improves efficiency through principal component analysis (PCA)-based dimensionality reduction and candidate pruning while applying Laplacian of Gaussian (LoG) filtering for illumination robustness. Despite these optimizations, the computational complexity still scales with the number of templates, and robustness under significant lighting variation remains limited. Additionally, the trade-off between accuracy and runtime performance limits its use in practical robotic systems.

In contrast, the present study overcomes these limitations by introducing a model-free, texture-free framework that does not depend on 3D CAD models of objects or template matching. The method instead leverages geometry-based features, such as surface normals and rim edges, which are derived from depth images and are inherently less sensitive to lighting conditions and texture variations. By structuring these features into uniform grids and using a transformer encoder to efficiently aggregate features, the approach avoids the scalability issues of template matching and reduces sensitivity to depth noise compared to prior RGB-D refinement pipelines. These improvements allow the proposed method to balance accuracy, robustness, and computational efficiency in real-world robotic applications involving tableware manipulation.

## 3. Methods

We propose a texture-free 6-DoF object pose estimation method based on transformer encoder architecture. The pipeline begins with object detection on a color image using a video foundation model. Specifically, we utilize state-of-the-art open-vocabulary object detectors such as Grounding DINO and YOLO-World, which enable robust and flexible detection of diverse tableware categories without requiring category-specific retraining. These models can generate accurate bounding boxes around tableware instances, including plates, bowls, and cups. This effectively localizes objects of interest within the scene and serves as the basis for subsequent segmentation and depth-based processing.

**Figure 2:**
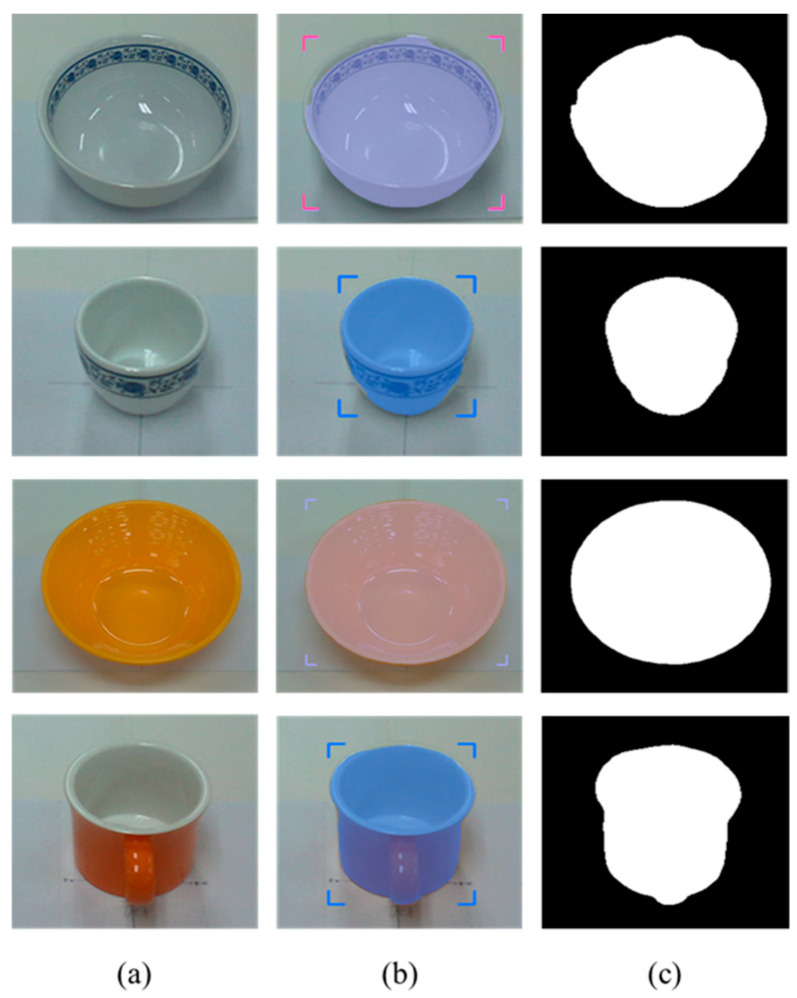
Tableware detection and mask generation using a video foundation model for object detection and segmentation: (**a**) input image of tableware, (**b**) detected bounding boxes, and (**c**) corresponding segmentation masks.

After detecting the target objects, we use the Segment Anything Model (SAM) to extract a precise binary mask for each one. This segmentation step is critical for ensuring accurate spatial localization. Following segmentation, the corresponding depth image is utilized to extract 3D surface vertices within the masked region. These vertices are then uniformly partitioned into grid cells, and a surface normal vector is computed for each cell. The resulting set of normal vectors captures local geometric features from both the surface and rim of the object. These features are subsequently integrated into a Transformer-based pose estimation network, which predicts the object’s 6-DoF pose in a texture-independent manner. Figure 2 illustrates the process in which tableware is detected from an image captured by the camera, and bounding boxes are extracted. These bounding boxes are then used to generate corresponding segmentation masks for each detected object.

Using the segmentation mask extracted from the color image, we isolate the region of interest in the corresponding depth image and extract the three-dimensional surface vertices of the tableware object. However, the number of vertices obtained from this process is inherently variable, as it depends on the pixel area of the mask, which is affected by both the object’s physical size and its distance from the camera. This variability gives rise to inconsistent input dimensions that are incompatible with standard deep learning architectures, which require fixed-size inputs for batch processing and efficient training.

To address this issue, the irregular vertex set is transformed into a structured, fixed-dimensional representation. First, a logical “AND” operation is applied to the object segmentation mask and the corresponding depth image. This isolates the depth values belonging exclusively to the target object within the two-dimensional image space. Then, depth values outside the mask are set to zero, and a rectangular 2D bounding box is used to bound the valid masked region.

Since the size of the masked region depends on the object’s distance from the camera, the region is normalized by dividing it into a fixed-resolution grid (e.g., 16 × 16 cells). Each grid cell contains dozens to hundreds of 3D vertices derived from the masked depth values. We compute the surface normal vector for each cell by applying singular value decomposition (SVD) to the local set of vertices. SVD captures the dominant orientation of the local surface patch, providing a robust estimate of the normal vector despite sensor noise or partial occlusion.

For each grid cell, the average 3D position (the centroid of the vertices) and the computed normal vector are aggregated to create a unified local feature. Using a 16 × 16 grid as an example, the input to the pose estimation model is a tensor of shape (16, 16, 6). The six dimensions of the feature vector correspond to the concatenated components of the surface normal vector and the average 3D position of each cell. This representation allows the pose estimation model to learn spatially organized geometric features with a consistent input size, regardless of the object’s scale or viewing distance.

The grid-based approach offers several advantages. Firstly, it enforces a fixed input size, thereby enabling the use of transformer encoders and other deep models without requiring additional padding or truncation strategies. Secondly, the aggregation of information within each grid cell enhances the representation’s robustness to sensor noise and local geometric variations, while preserving sufficient spatial structure to support accurate pose inference. Moreover, the grid resolution can be adjusted to strike a balance between spatial precision and computational efficiency. However, aggregating multiple point clouds into a single representative vector per cell may lead to the loss of fine-grained surface details, particularly in regions of small or complex objects. The quality of the resulting representation depends on the density and uniformity of the original point cloud within the segmented area. Nevertheless, we find that this strategy provides a practical compromise between structural fidelity and model compatibility. It generalizes well to objects of varying sizes and viewing distances and maintains consistent performance across a wide range of real-world scenarios.

**Figure 3:**
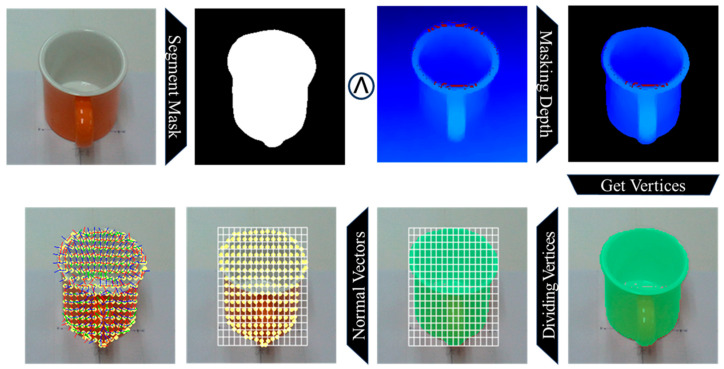
The procedure for partitioning the surface vertices of the tableware object into grid cells of uniform spacing. First, a segmentation mask obtained from an RGB image is applied to the depth image to extract surface vertices. Then, the vertices are uniformly divided into a 2D grid. Finally, the center position and surface normal vector are calculated for each grid cell to generate a fixed-size feature representation for pose estimation.

**Figure 4:**
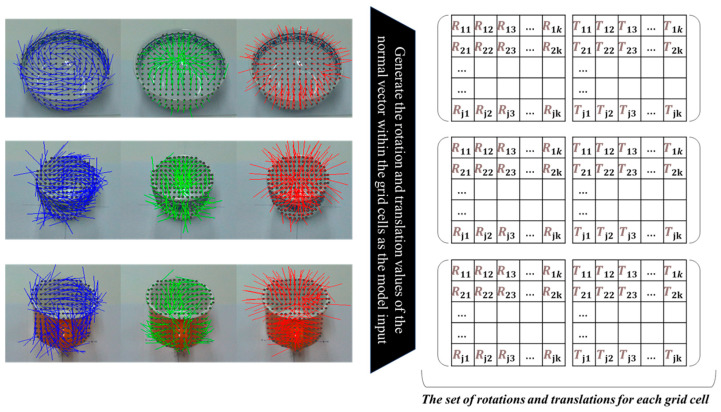
The procedure of calculating the surface normal vectors from the vertices in each grid cell and constructing the corresponding input features for the pose estimation model. Each row shows a different object, such as a bowl or cup, with its grid-partitioned vertices and computed surface normal vectors visualized from multiple viewpoints.

Figure 3 illustrates the process of dividing the surface vertices of the tableware object into uniformly spaced grid cells. Figure 4 presents the subsequent computation of normal vectors for the vertices within each grid cell and the generation of input features for the pose estimation model.

A distinguishing geometric characteristic of tableware objects is the presence of an open upper rim, a feature commonly shared across categories such as plates, bowls, and cups. The rim provides a stable structural reference that can mitigate pose estimation errors caused by occlusion or deformation due to food inside the container. The normal vectors of the rim’s edge vertices tend to align in a consistent direction when the object is placed on a table. Therefore, rim normals exert a dominant influence on pose estimation, providing reliable information for determining the object’s central axis and rotational orientation.

To explicitly incorporate this property, rim-specific vertices are extracted from the segmentation mask by identifying the outer contour of the tableware. From this contour, the leftmost, rightmost, and topmost points in image space are selected, which are assumed to correspond to the upper rim region. Using these points, the approximate spatial area of the rim is localized, and the corresponding three-dimensional vertices are obtained from the depth image. The surface normals of these rim vertices are then computed to yield robust constraints for estimating the overall pose.

In contrast, the other surface vertices have a smaller but complementary role. They partially contribute to the pose estimation and provide information about the object’s shape and scale. This helps the model distinguish between different categories of tableware, such as differentiating between a shallow plate and a deep bowl, even when texture information is absent. The proposed method combines rim normals with general surface features to incorporate both dominant geometric priors and discriminative features. This enables robust 6-DoF pose estimation across diverse tableware types.

**Figure 5:**
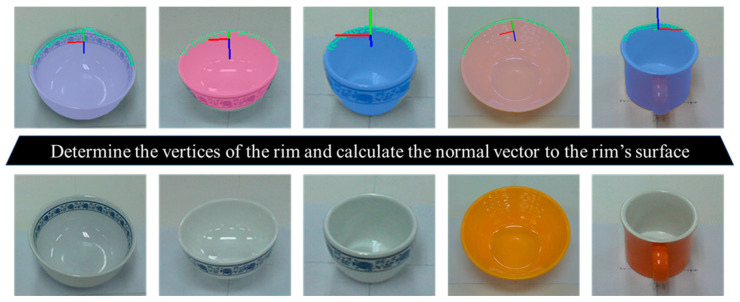
Visualization of the upper rim vertices and their corresponding normal vectors.

Figure 5 shows the vertices and normal vectors of the upper rim of a tableware object, extracted based on the object’s outer contour.

The pose estimation model consists of transformer encoders, followed by a multi-layer perceptron (MLP) head. The transformer encoders process a set of input features, such as surface normals and spatial coordinates, to capture both local and global geometric relationships across an object’s surface. After encoding these spatial features, the transformer outputs a fixed-length feature vector that encapsulates the aggregated geometric information. The MLP then maps this feature vector to a structured output that represents the object’s three-dimensional rotation, translation, size, and category ID. This architecture enables the model to infer multiple pose-related parameters jointly in an end-to-end learning framework.

To effectively process high-dimensional geometric inputs derived from grid-based representations, our proposed pose estimation model incorporates several architectural improvements to the conventional transformer encoder. The motivation for these modifications can be traced back to three key areas: parallel multi-object poses inference, parameter efficiency, and training stability. These modifications draw inspiration from prior ablation research [33].

The first element to consider is the expansion of output dimension of the multi-head attention layer for parallel pose prediction. The design of conventional transformer encoders is generally oriented around the generation of a solitary output vector for each input token. However, in our scenario, each input instance may contain multiple object candidates (e.g., several pieces of tableware in a single image frame). By structuring the output to support multiple object-specific embeddings, the model can generate multiple pose predictions in parallel within a single forward pass. This approach enhances computational efficiency in real-time applications, including multi-object tabletop scene analysis, and eliminates the necessity for iterative object-wise inference.

Secondly, the reduction in parameters is achieved through the implementation of low-rank adaptation in attention layers. Modern transformer models are often over-parameterized, especially in attention layers where large fully connected dense layers contribute significantly to memory usage and training time. To address this issue, we integrate low-rank weight decomposition [34] into the attention mechanism. Specifically, weight matrices in the dense layers are factorized into lower-rank representations, thereby enabling the approximation of high-dimensional projections with a significantly reduced number of parameters. This decomposition strategy reduces the number of trainable weights without sacrificing expressiveness, thereby lowering memory requirements, and accelerating both training and inference. Empirically, we observe that this approach maintains competitive accuracy compared to the full-rank baseline while offering better generalization, particularly in data-limited settings.

Thirdly, enhancements have been made to both the normalization and activation functions. To enhance the convergence and stability of the training process, the conventional Layer Normalization procedure is substituted with Root Mean Square (RMS) Normalization [35]. RMSNorm normalizes inputs based on the root mean square of feature activations rather than mean-variance statistics. This approach offers enhanced robustness to input distribution shifts and improved compatibility with deeper transformer stacks. Additionally, the Swish (SiLU) activation function [36] is adopted in place of ReLU or GELU [37] in the feedforward layers. Swish is a smooth, non-monotonic function that has been demonstrated to enhance gradient flow and performance in deep networks. In the experiments conducted, this modification consistently yielded enhancements in training stability and final pose estimation accuracy, particularly in the context of learning subtle geometric patterns from normal vector fields.

**Figure 6:**
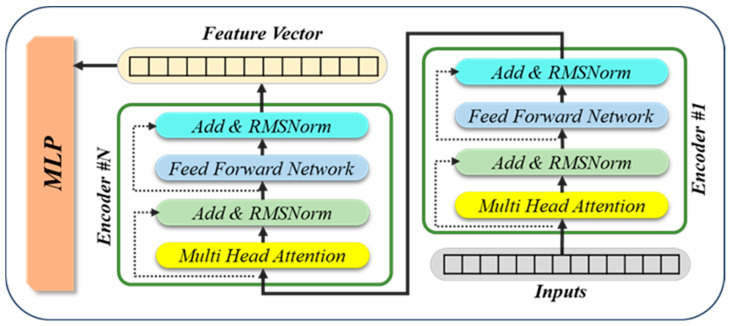
A schematic diagram of the enhanced transformer encoder combined with an MLP for poses estimation.

As demonstrated in Figure 6, the architecture of the enhanced transformer encoder integrated with an MLP head for 6-DoF pose estimation is illustrated.

To quantitatively assess the performance of pose estimation for tableware objects, an appropriate evaluation metric is required. In this study, we perform model-free pose estimation, which does not rely on 3D CAD models of the objects. Consequently, conventional metrics widely used for 6D object pose estimation, such as the Average Distance of Model Points (ADD) or the ADD-S metric for symmetric objects, cannot be applied.

Instead, we evaluate the system using pose-parameter-based errors. Specifically, we compute the rotation error and translation error between the estimated pose and the ground truth. Additionally, we introduce a reprojection-based metric. We use the 3D bounding box corner points of each object are used as keypoints, which are then projected onto the 2D image plane using the camera intrinsics. The reprojection error is calculated as the average distance between the projected estimated keypoints and the ground-truth projections. Together, these metrics provide a comprehensive evaluation of pose accuracy without requiring access to object’s CAD models.

The rotation error $\theta_{err}$ between the predicted rotation matrix $\hat{R}\in SO(3)$ and ground-truth rotation matrix R∈SO(3) is defined as the geodesic distance on the rotation group SO(3), as shown in Equation (1). This formulation yields the angular difference in radians or degrees between the two rotations, and it is independent of the chosen coordinate system. A lower value indicates closer alignment between the estimated and ground-truth orientations.

(1) $$\theta_{err}=arccos\left(\frac{trace\left(R^{-1}\hat{R}\right)-1}{2}\right)$$

The translation error $e_{trans}$ evaluates the Euclidean distance between the estimated translation vector $\hat{t}\in R^{3}$ and the ground-truth translation vector $t\in R^{3}$. It is defined as the L2-Norm difference between the two vectors, as in Equation (2). This metric reflects the discrepancy in position in 3D space between the estimated and ground-truth object centroids.

(2) $$e_{trans}={\left\|\hat{t}-t\right\|}_{2}$$

To account for the impact of pose estimation accuracy on image-space alignment, we additionally compute the reprojection error. Let $Ρ=\left\{p_{i}\in R^{3}|i=1,\ldots ,N\right\}$ denote the set of 3D keypoints, chosen from the eight corner points of the object’s 3D bounding box. For each keypoint in Ρ, we obtain its estimated and ground-truth 2D projections onto the image plane are obtained using the camera intrinsic matrix K. The reprojection error $e_{reproj}$ is computed as the mean L2 distance between the estimated and ground-truth projections of all N keypoints in Equation (3), where $\pi \left(x\right)=\left(\frac{f_{x}x}{z}+c_{x},\;\frac{f_{y}y}{z}+c_{y}\right)$ is the projection function defined by the camera intrinsics $\left(f_{x},{\;f}_{y},\;{\;c}_{x},{\;c}_{y}\right)$.

This metric reflects how accurately the estimated pose preserves the visual alignment of the object’s keypoints in the image plane.

(3) $${\hat{u}}_{i}=\pi \left(\hat{R}p_{i}+\hat{t}\right),\;\;u_{i}=\pi \left(Rp_{i}+t\right),\;e_{reproj}=\frac{1}{N}\sum_{i=1}^{N}{\left\|\hat{u_{i}}-u_{i}\right\|}_{2}$$

## 4. Experiments

To evaluate pose estimation, we created a dataset consisting of ten types of tableware, including bowls, cups, and plates of various sizes. Each object was placed on a table and two ARUCO [38] markers were attached to either side to obtain accurate pose measurements. To ensure the reliability of the ground-truth pose information, we verified the recognition status of the markers and confirmed that the three-dimensional coordinates of the marker corner points transformed from the depth image were valid. We computed the final ground-truth pose of each object by averaging the poses of the two markers and adjusting for the height offset specific to each object.

The training dataset was created by placing tableware objects on a marker board and capturing images from various camera distances and viewpoints. Each image was preprocessed to extract the relevant geometric information of the objects, and the final dataset consisted of approximately 10,000 samples per object, resulting in a total of 100,000 samples used for training. Additionally, we constructed a validation dataset of about 50,000 samples and a test dataset of about 3000 samples were also constructed to evaluate the performance and generalization of the proposed model.

To enhance robustness and improve generalization, several data augmentation strategies were employed. First, random noise was added to the depth images during preprocessing to simulate environmental variability and sensor imperfections. Second, random masking was applied to portions of the grid-based model input during training to create artificial occlusion scenarios. This procedure encouraged the model to infer object poses reliably even under partial visibility. Through the design of this dataset and its augmentation, the training process captured a wide range of viewing conditions, noise levels, and cases of occlusion, ensuring that the pose estimation model could generalize effectively to unstructured, real-world environments.

The transformer encoder used in the pose estimation model had 256 units per attention layer and 16 attention heads in each encoder block. This structure was repeated eight times, yielding a total of approximately 9.66 million trainable parameters across the entire model.

The experiments were conducted using an Intel RealSense L515 LiDAR depth camera, which can capture depth data within a range of 0.25 to 9 m. The device has a 2-megapixel RGB sensor that supports up to 1920 × 1080 resolution at 30 frames per second and a depth sensor that provides up to 1024 × 768 resolution at 30 frames per second. For our experiments, we captured RGB images at a resolution of 1280 × 720 and depth images at a resolution of 1024 × 768. To ensure spatial correspondence, we aligned the depth images to the RGB images using the RealSense API. The camera has horizontal and vertical fields of view of approximately 70° × 55° and achieves high depth accuracy. The average error is less than 5 mm at 1 m and less than 14 mm at 9 m when measured at VGA resolution under 95% reflectivity conditions.

The parameters of the transformer encoder in the pose estimation model and the camera used in the experiment are summarized in Table 1 below.

To capture a variety of realistic conditions, we moved the camera around the objects along irregular trajectories (left, right, up, and down) at distances ranging from 0.3 to 1.2 m. We acquired 300 color and depth image pairs for each object, resulting in a dataset that covers diverse viewpoints and scales.

In terms of perception performance, we compared two open-vocabulary detectors, YOLO-World and Grounding DINO. Grounding DINO performed better for vocabularies such as “dish,” “plate,” and “cup.” However, Grounding DINO required more than 700 milliseconds per inference in our real-world demonstration scenarios, which limited its practicality. The lower performance of the hardware platform equipped with an NVIDIA RTX 2060 mobile GPU, which was used for the demonstration, also constrained the use of computationally heavy models like Grounding DINO. Therefore, we primarily adopted YOLO-World in our system. The experimental results reported in the manuscript are based on YOLO-World and achieved an average inference time of 100 milliseconds. However, YOLO-World occasionally failed to detect tableware or misclassified other objects, resulting in an overall detection success rate of 86% for tableware objects.

Additionally, segmentation occasionally produced imperfect boundaries, such as masks that extended slightly beyond the true edges of objects. These cases were reflected in the generation of the training dataset and did not adversely affect the inference performance of the pose estimation model.

**Figure 7:**
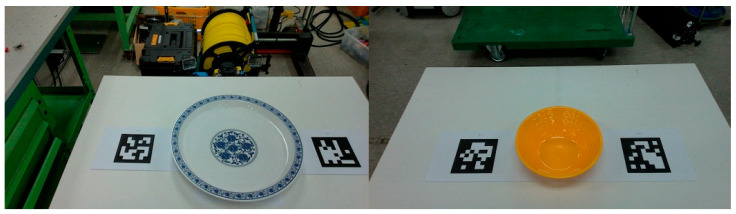
Experimental setup for collecting validation data to measure pose estimation error of individual tableware objects.

Figure 7 shows the data acquisition setup used to generate the validation dataset for the tableware pose estimation task.

The results of the average pose error between the ground-truth poses obtained from the markers and the predicted poses of the tableware, computed over 300 samples for each object in the validation image set, are summarized in Table 2 below.

**Figure 8:**
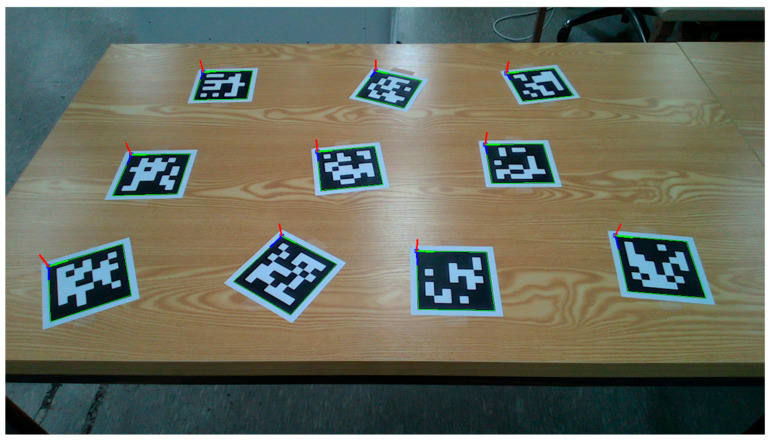
Arrangement of markers for ground-truth extraction in the validation of multi-object tableware object pose estimation.

**Figure 9:**
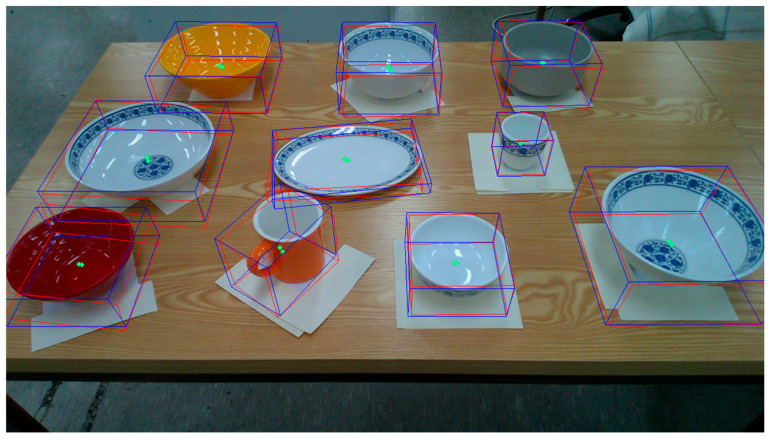
Execution of pose inference by the estimation model on all tableware objects.

To evaluate the estimation model’s ability to simultaneously estimate the poses of multiple objects, we conducted an experiment in which we placed 10 ARUCO markers arbitrarily on a table. Then, we computed the ground-truth poses based on the markers’ spatial configuration, as shown in Figure 8. Next, as shown in Figure 9, we positioned a set of tableware objects on top of the marker centroids and performed pose inference using the trained estimation model.

The camera was positioned approximately 1.2 m from the objects at a fixed location and 300 color and depth images were captured. Although the camera remained stationary, the captured images were not identical due to variations in illumination and inherent noise in the depth sensor, which introduced subtle differences across frames.

We computed the average pose error for each object by comparing the inferred pose with the marker-based ground truth. We also measured the below-threshold ratio, which is the percentage of the total number of frames in which the estimation error fell below a specified threshold. The results are summarized in Table 3.

Consequently, we observed larger pose errors for tableware objects positioned near the edges of the image frame. We assumed this was due to limitations in the training dataset. Objects at the edge of the image were not assigned accurate target values, which resulted in poor learning performance in those regions.

In a demonstration scenario involving the collection of tableware, the pose estimation system successfully recognized the poses of multiple tableware objects within an acceptable error margin for robotic manipulation, enabling the robot to grasp and retrieve the items accurately. Experiments were conducted using a Neuromeka Indy7 [39] collaborative robot, which is a 7-axis manipulator with a payload capacity of 7 kg and a maximum reach of 800 mm. It provides a high repeatability of 0.03 mm and a maximum joint speed of 815 degrees/s, ensuring precise and efficient operation. In addition, the Indy7 is equipped with collision detection and an IP67-rated dustproof and waterproof design, which enhances safety and reliability during manipulation tasks in real-world environments. The specifications of the robot arm used in the experiment are summarized in Table 4.

**Figure 10:**
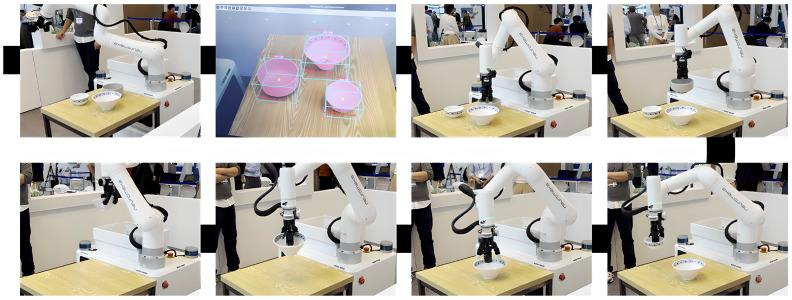
Tableware detection and retrieval process performed by the collection robot in the demonstration scenario.

The integrated mobile robot system, which combines perception and manipulation, achieved an 84% grasping success rate, demonstrating the practical effectiveness of the proposed approach in real-world conditions. Figure 10 illustrates the execution of this process, showing the system performing pose-based recognition and collection of tableware using a robotic arm.

## 5. Discussion

This paper presents a method for model-free and texture-free, six-degree-of-freedom (6-DoF) pose estimation of tableware objects using depth-based geometric features and a transformer encoder architecture. The proposed approach generates a fixed-size input representation by partitioning the three-dimensional surface vertices extracted from a depth image into a uniform grid. Then, it computes the mean positions and surface normal vectors within each cell. This structure enables consistent input formatting while preserving geometric information.

The system uses either YOLO-World or Grounding DINO as object detectors and employs the Segment Anything Model (SAM) to generate segmentation masks. To improve robustness, especially for objects with an open top such as plates, bowls, and cups, additional features are extracted from the upper rim based on contour information.

Several modifications were made to the transformer encoder architecture. The output dimension of the multi-head attention was expanded to support multi-object inference. Low-rank weight decomposition was applied to reduce the number of parameters. The layer normalization and activation components were replaced with RMS normalization and Swish function, respectively. These changes aim to enable efficient learning and stable training on geometric inputs.

Experimental validation was conducted on a dataset of 10 tableware types under varying viewpoints and distances. The results showed that the system achieved pose estimation accuracy within acceptable bounds for manipulation tasks. In multi-object scenarios, performance remained consistent, although higher errors were observed near the image periphery due to limitations in training coverage. Additional evaluation using the below-threshold ratio metric confirmed the system’s reliability under depth sensor noise and illumination variation.

In a demonstration environment, the pose estimation system was used for a tableware collection task that involved a mobile robot equipped with a manipulator. The robot could detect and estimate the poses of multiple tableware objects and then collect them based on the inferred poses. These results suggest that the proposed method can be applied to real-world scenarios with limited texture information.

Although the proposed pose estimation and grasping system achieved an overall grasp success rate of approximately 84% in a real demonstration environment, several failure cases were observed during the demonstration trials. Analysis of these failures reveals a set of common factors related to perception, pose estimation, and manipulation.

The tableware pose estimation pipeline begins with object detection and segmentation using video foundation models, such as YOLO-World, Grounding DINO, and SAM. If this stage fails, either due to complete detection failure or inaccurate segmentation, the subsequent pose estimation process cannot proceed. In practice, segmentation masks sometimes exceed true object boundaries or omit portions of the rim. This reduces the reliability of rim-based normal vectors, which are critical for pose estimation. Therefore, the performance of the video foundation model has a significant impact on the overall system accuracy, emphasizing the importance of using cutting-edge detection and segmentation methods to maximize the success rate of the first stage.

The Intel RealSense L515 depth camera often produced inaccurate or incomplete depth measurements, particularly for tableware with red or black surfaces, which absorb the infrared laser used for depth sensing. These distortions negatively impacted vertex extraction and subsequent normal vector estimation. Furthermore, desynchronization between the RGB and depth streams was observed during rapid camera motion, which degraded the quality of the generated vertices and reduced pose estimation accuracy. These limitations suggest the need for faster image acquisition and synchronized capture via the camera API to ensure consistent RGB-D alignment.

A significant portion of the total inference time was consumed during the preprocessing stage, particularly when computing surface normals from vertices via SVD. Preprocessing accounted for approximately 80% of the total runtime, while object detection and segmentation, as well as transformer-based pose inference, each accounted for 10%. With the current grid size of 16 × 16, preprocessing required an average of 0.8 s per object. Although increasing the grid resolution to 32 × 32 could provide more detailed surface features and potentially improve pose accuracy, it would increase the average preprocessing time to over four seconds, making it impractical for real-time applications. Therefore, accelerating normal vector computation through GPU parallelization or algorithmic optimization is essential to improving system throughput.

The system demonstrated higher pose estimation errors for objects positioned near the periphery of the camera’s field of view. This limitation arises from both reduced depth accuracy at the image edges and insufficient training data representing peripheral placements. Additionally, the model was not sufficiently trained on occlusion scenarios, such as partially overlapping tableware, which further contributed to pose estimation errors in cluttered scenes. To improve generalization in unseen conditions, the model requires additional training with more diverse camera viewpoints, distances, and occlusion cases.

Future work will focus on improving the reliability and efficiency of the pose estimation system. To reduce early-stage errors, detection and segmentation should be enhanced using state-of-the-art video foundation models. To overcome computational bottlenecks and enable real-time operation, we will explore GPU-based parallelization and adaptive grid partitioning. Additionally, we will expand the training dataset to include more diverse scenarios, such as occlusions and peripheral placements, to improve the model’s generalization capability.

## 6. Conclusions

This study presents a framework that does not rely on computer-aided design (CAD) models or texture information to estimate the six-degree-of-freedom (6-DoF) pose of tableware objects. This approach overcomes the limitations of conventional methods that depend on surface texture information or CAD models. The proposed approach uses geometry-based features, such as surface normals from grid-partitioned vertices and rim-edge information. The framework uses a transformer encoder architecture to accurately and efficiently infer pose.

Through experiments on ten categories of tableware, the method demonstrated robust performance, achieving average rotational and translational errors within practical tolerances for robotic manipulation. Additionally, deployment on a mobile robot equipped with a manipulator confirmed the method’s real-world applicability by achieving an 84% grasping success rate in demonstration scenarios.

In contrast to prior methods, which are often limited by sensitivity to depth noise, scalability issues in template matching, or reliance on detailed 3D models, the proposed framework offers a computationally efficient and generalizable solution for textureless objects. Although some limitations remain, such as dependency on the accuracy of the detection and segmentation models, poor performance near image borders, and difficulty with severe occlusion, this study establishes a strong foundation for autonomous tableware handling in restaurant environments.

Future research will focus on accelerating the computation of normal vectors for real-time deployment and expanding the training dataset to include a wider range of viewpoints, occlusion scenarios, and object categories. These improvements will make the proposed framework more robust, scalable, and applicable to practical service robotics.

## Figures and Tables

**Table 1:** Specifications of the transformer encoder in the pose estimation model and camera.

Component	Parameter	Value/Specification
Transformer Encoder Model	Attention units per layer	256
	Attention heads per encoder block	16
	Number of encoder blocks	8
	Total trainable parameters	~9.66 million
Camera (Intel RealSense L515)	RGB sensor resolution	1280 × 720
	Depth sensor resolution	1024 × 768
	Alignment	Depth aligned to RGB using RealSense API
	Field of view (H × V)	~70° × 55°
	Depth accuracy	<5 mm @ 1 m; <14 mm @ 9 m (VGA, 95% reflectivity)

**Table 2:** The results of the pose error and the process time for each tableware object.

Tableware Objects	Rotation Error [deg] (Mean ± Std)	Translation Error [mm] (Mean ± Std)	Reprojection Error [mm] (Mean ± Std)	Process Time [s] (Mean)
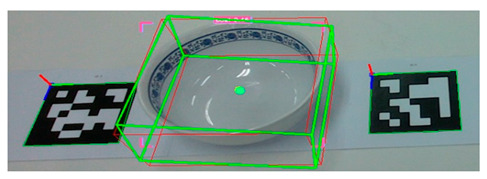 ID: 02, Type: Bowl	3.97 ± 2.65	16.09 ± 5.96	17.22 ± 6.04	0.699
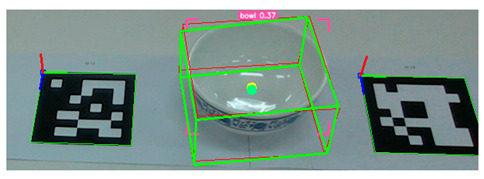 ID: 03, Type: Bowl	4.34 ± 2.14	14.23 ± 5.62	15.12 ± 5.21	0.670
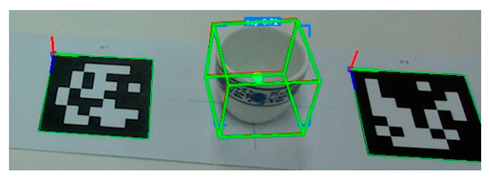 ID: 05, Type: Cup	2.82 ± 1.88	5.05 ± 2.54	5.70 ± 2.54	0.624
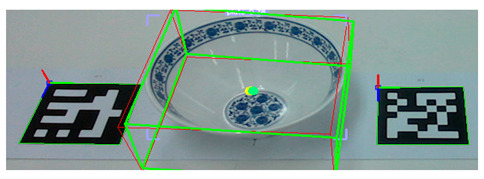 ID: 10, Type: Bowl	3.18 ± 1.83	14.24 ± 4.70	15.19 ± 4.69	0.692
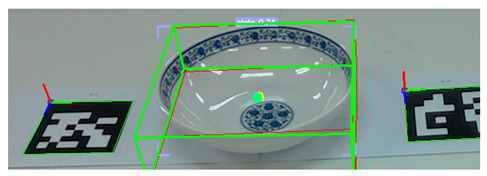 ID: 12, Type: Bowl	4.47 ± 1.60	20.43 ± 6.20	21.08 ± 5.94	0.852
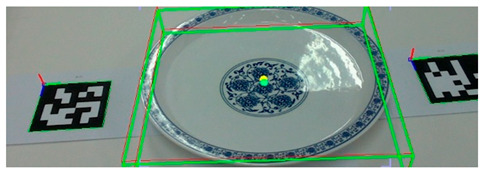 ID: 18, Type: Plate	2.55 ± 1.64	16.04 ± 5.19	17.44 ± 5.88	1.243
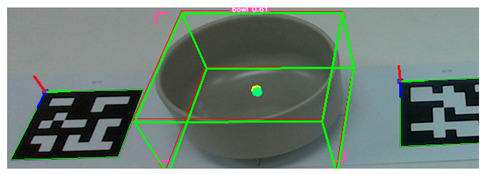 ID: 21, Type: Bowl	3.12 ± 1.81	12.54 ± 4.69	13.26 ± 4.75	0.788
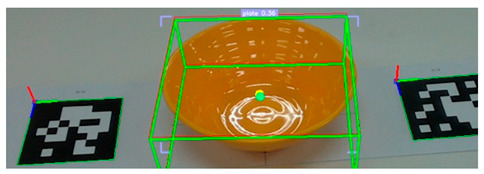 ID: 23, Type: Bowl	3.25 ± 1.59	15.66 ± 4.31	16.56 ± 4.24	0.852
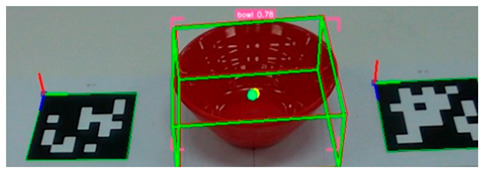 ID: 24, Type: Bowl	3.28 ± 1.45	14.15 ± 4.53	14.74 ± 4.34	0.721
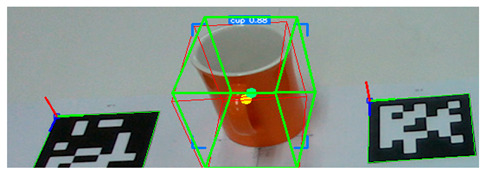 ID: 27, Type: Cup	4.35 ± 2.35	7.16 ± 3.63	9.07 ± 3.67	0.679
Average	3.53 ± 1.90	13.56 ± 4.74	14.54 ± 4.73	0.782

**Table 3:** Results for pose estimation error and below-threshold ratio for tableware objects.

Object ID	Rotation Error [deg] (Mean ± Std)	Translation Error [mm] (Mean ± Std)	Below-Threshold Ratio [%]		
			≤5° 5 cm	≤10° 2 cm	≤10° 5 cm
02	6.53 ± 2.60	8.61 ± 2.59	25.1	90.2	90.2
03	6.97 ± 2.65	9.27 ± 2.35	24.6	61.5	88.8
05	7.07 ± 2.65	9.11 ± 2.35	26.8	91.1	91.1
10	6.80 ± 2.52	8.68 ± 2.51	21.8	88.9	88.9
12	7.41 ± 2.52	12.40 ± 4.19	17.2	55.9	71.4
16	6.72 ± 2.83	9.04 ± 3.94	22.6	76.4	83.3
21	6.85 ± 2.41	8.32 ± 2.67	26.0	91.1	91.1
23	7.13 ± 2.45	9.40 ± 2.72	23.4	87.7	87.7
24	7.17 ± 2.70	8.42 ± 2.57	29.8	92.3	92.3
27	5.99 ± 1.70	5.19 ± 2.39	57.4	100.0	100.0

**Table 4:** Specifications of the collaborative robot arm.

Component	Parameter	Value/Specification
Collaborative Robot Arm (Neuromeka Indy7)	Degrees of freedom	7-axis
	Payload capacity	7 kg
	Maximum reach	800 mm
	Repeatability	0.03 mm
	Maximum joint speed	815°/s
	Safety features	Collision detection, IP67 dustproof and waterproof

## Data Availability

Data is contained within the article.
